# Endocannabinoids mediate muscarine-induced synaptic depression at the vertebrate neuromuscular junction

**DOI:** 10.1111/j.1460-9568.2007.05422.x

**Published:** 2007-03-01

**Authors:** Zachary Newman, Priya Malik, Tse-Yu Wu, Christopher Ochoa, Nayantara Watsa, Clark Lindgren

**Affiliations:** Department of Biology, Grinnell College Grinnell, IA 50112, USA

**Keywords:** Anolis carolinensis, endocannabinoids, muscarinic, neuromuscular junction, synaptic depression

## Abstract

Endocannabinoids (eCBs) inhibit neurotransmitter release throughout the central nervous system. Using the *Ceratomandibularis* muscle from the lizard *Anolis carolinensis* we asked whether eCBs play a similar role at the vertebrate neuromuscular junction. We report here that the CB_1_ cannabinoid receptor is concentrated on motor terminals and that eCBs mediate the inhibition of neurotransmitter release induced by the activation of M_3_ muscarinic acetylcholine (ACh) receptors. *N*-(piperidin-1-yl)-5-(4-iodophenyl)-1-(2,4-dichlorophenyl)-4-methyl-1H-pyrazole-3-carboxamide, a CB_1_ antagonist, prevents muscarine from inhibiting release and arachidonylcyclopropylamide (ACPA), a CB_1_ receptor agonist, mimics M_3_ activation and occludes the effect of muscarine. As for its mechanism of action, ACPA reduces the action-potential-evoked calcium transient in the nerve terminal and this decrease is more than sufficient to account for the observed inhibition of neurotransmitter release. Similar to muscarine, the inhibition of synaptic transmission by ACPA requires nitric oxide, acting via the synthesis of cGMP and the activation of cGMP-dependent protein kinase. 2-Arachidonoylglycerol (2-AG) is responsible for the majority of the effects of eCB as inhibitors of phospholipase C and diacylglycerol lipase, two enzymes responsible for synthesis of 2-AG, significantly limit muscarine-induced inhibition of neurotransmitter release. Lastly, the injection of (5Z,8Z,11Z,14Z)-*N*-(4-hydroxy-2-methylphenyl)-5,8,11,14-eicosatetraenamide (an inhibitor of eCB transport) into the muscle prevents muscarine, but not ACPA, from inhibiting ACh release. These results collectively lead to a model of the vertebrate neuromuscular junction whereby 2-AG mediates the muscarine-induced inhibition of ACh release. To demonstrate the physiological relevance of this model we show that the CB_1_ antagonist *N*-(piperidin-1-yl)-5-(4-iodophenyl)-1-(2,4-dichlorophenyl)-4-methyl-1H-pyrazole-3-carboxamide prevents synaptic inhibition induced by 20 min of 1-Hz stimulation.

## Introduction

Cannabinoids, the active ingredients found in the marijuana plant *Cannabis sativa* (Ameri, 1999; Martin *et al.*, 1999), produce their biological effects through binding to specific G-protein-coupled receptors (Howlett *et al.*, 2002). The term endocannabinoid (eCB) refers to endogenously released compounds that alter function by binding to these receptors (Devane *et al.*, 1992; Evans *et al.*, 1992). The eCBs are synthesized *de novo* from membrane phospholipids and are released through an unknown mechanism (Freund *et al.*, 2003; but see Ronesi *et al.*, 2004). Recently, eCBs have been shown to act as retrograde signalling molecules in several areas of the central nervous system (for reviews see Kreitzer & Regehr, 2002; Wilson & Nicoll, 2002). Depolarization of the postsynaptic neurone and the resulting elevation of intracellular Ca^2+^ triggers eCB release (Ohno-Shosaku *et al.*, 2001; Wilson & Nicoll, 2001; Brenowitz & Regehr, 2003). The activation of muscarinic acetylcholine (ACh) receptors (mAChRs) (Kim *et al.*, 2002; Ohno-Shosaku *et al.*, 2003; Fukudome *et al.*, 2004) can also trigger the release of eCBs. The eCBs released in neural tissue usually bind to the CB_1_ receptor subtype and inhibit the release of neurotransmitter from the presynaptic terminal (Kreitzer & Regehr, 2001, 2002; Maejima *et al.*, 2001; Ohno-Shosaku *et al.*, 2001; Wilson & Nicoll, 2001; Diana *et al.*, 2002; Yoshida *et al.*, 2002; but see Van Sickle *et al.*, 2005).

The inhibition of neurotransmitter release via the activation of mAChRs has been observed throughout both the central and peripheral nervous systems (for reviews, see Starke *et al.*, 1989; Caulfield, 1993; Boehm & Huck, 1997). It has been well established that activation of mAChRs at the vertebrate neuromuscular junction (NMJ) modulates the release of the neurotransmitter ACh (Ganguly & Das, 1979; Duncan & Publicover, 1979; Michaelson *et al.*, 1979; Standaert, 1982; Wali *et al.*, 1988; Slutsky *et al.*, 1999, 2001; Minic *et al.*, 2002). In particular, activation of the M_1_ subtype of the mAChR enhances ACh release (Slutsky *et al.*, 1999; Graves *et al.*, 2004), whereas activation of the M_2_ and/or M_3_ subtype inhibits release (Slutsky *et al.*, 1999, 2001; Graves *et al.*, 2004). Recently, the M_1_-mediated enhancement and the M_3_-mediated inhibition of neurotransmitter release at the lizard NMJ have been shown to require the synthesis and extracellular diffusion of nitric oxide (NO) (Graves *et al.*, 2004).

As eCBs mediate the suppression of neurotransmitter release induced by M_1_ and M_3_ receptor activation in the hippocampus (Fukudome *et al.*, 2004), we searched for a similar involvement of eCBs at the lizard NMJ. Using immunofluorescence, we localized CB_1_ receptors to the NMJ and, using physiological and pharmacological approaches, discovered that eCBs [primarily 2-arachidonoylglycerol (2-AG)] do indeed mediate the depression of neurotransmitter release induced by the activation of M_3_ mAChRs. Furthermore, this depression requires NO, acting via cGMP and cGMP-dependent protein kinase, involves a decrease in the size of the calcium transient in the presynaptic nerve terminal, and requires an eCB transporter in the muscle membrane. Lastly, we demonstrate the physiological relevance of eCBs by showing that a form of long-term synaptic depression requires functional CB_1_ receptors.

## Materials and methods

### Experimental preparation and solutions

**Fig. 8:**
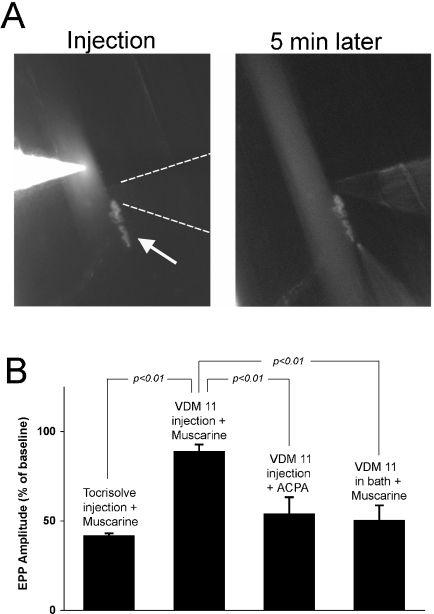
Intracellular injection of cannabinoid transport inhibitor (5Z,8Z,11Z,14Z)-*N*-(4-hydroxy-2-methylphenyl)-5,8,11,14-eicosatetraenamide (VDM 11) into the muscle blocks muscarine-induced depression. (A) Micrographs showing the injection of VDM 11 into a muscle fibre near an end plate. The image on the left was collected during the pressure injection of VDM 11 and rhodamine B. The neuromuscular junction (NMJ) was stained with tetraethylrhodamine-α-bungarotoxin and can be seen just below and to the right of the injection electrode (arrow). A faint image of the recording electrode can also be seen approaching from the right (highlighted by dashed lines). The image on the right was collected 5 min after injecting the muscle fibre. The injection electrode has been removed from the field of view and the extracellular pipette used to apply muscarine has been moved into position just below the neuromuscular junction. (B) Mean percent reduction of end-plate potential (EPP) amplitudes following the local application of muscarine or arachidonylcyclopropylamide (ACPA). Muscarine was applied to NMJs injected with the solvent Tocrisolve® (*n* = 3) or with VDM 11 dissolved in Tocrisolve® (*n* = 6). ACPA was applied to NMJs injected with VDM 11 (*n* = 8). Muscarine was also applied to NMJs bathed in VDM 11 (*n* = 4). The Student's *t*-test was used to calculate the statistical significance of the differences between the indicated pairs of means.

Prior to being pithed, lizards (*Anolis carolinensis*; Carolina Biological Supply Co.) were placed at 7–10 °C for 8–10 min to facilitate the quick and accurate ablation of the forebrain. The ceratomandibularis muscle (and its associated nerve) was isolated from small lizards as described by Lindgren & Moore (1989) and pinned down in a Sylgard®-coated chamber containing fresh physiological saline solution composed of 158 mm NaCl, 2 mm KCl, 2 mm MgCl_2_, 5 mm HEPES, 2 mm CaCl_2_ and 2 g/L dextrose (pH adjusted to 7.3 using 1 m NaOH). Evoked end-plate potentials (EPPs) were reduced below the action potential threshold of the muscle by applying 10 µm d-tubocurarine chloride. For experiments indicated in Fig. 8, 2.5 µm d-tubocurarine chloride was used together with 1 µg/mL tetraethylrhodamine-α-bungarotoxin The procedures described above were approved by the Institutional Animal Use and Care Committee at Grinnell College.

In all of the experiments except the one described in Fig. 8, drugs were administered via the physiological saline solution bathing the preparation. Unless indicated otherwise, concentrated stock solutions of the various drugs were prepared in advance and frozen at −20 °C. On the day of the experiment, aliquots were diluted in physiological saline solution to their final concentrations. In the case of arachidonylcyclopropylamide (ACPA) or (5Z,8Z,11Z,14Z)-*N*-(4-hydroxy-2-methylphenyl)-5,8,11,14-eicosatetraenamide (VDM 11), the drug was obtained in Tocrisolve® (a soy oil and water emulsion) and diluted directly into physiological saline. In experiments where ACPA or VDM 11 was applied, the control solution contained Tocrisolve® at the same concentration as in the experimental solution.

For the experiments depicted in Fig. 8, muscarine or ACPA was applied locally to an identified NMJ through a glass pipette with a diameter of approximately 1 µm via back pressure applied with a pneumatic pico pump (PV 830; World Precision Instruments, Sarasota, FL, USA). Between one and six 2- and 5-s pressure pulses (10 s apart) were applied at 10–15 p.s.i. The electrode was filled with 20 µm muscarine or ACPA and either rhodamine B or fluorescein. The latter were used to track the dispersion of the pipette contents. The electrode was positioned within 100–200 µm of nerve terminals on the top surface of the muscle and the dispersion of the dye always enveloped the NMJ. Although we do not know the local concentration of muscarine or ACPA at the synapse, the concentrations used produced changes similar to bath application of either chemical.

2-(4-carboxyphenyl)-4,4,5,5-tetramethylimidazoline-1-oxyl-3-oxide potassium salt (carboxy-PTIO) was purchased from Molecular Probes (Eugene, OR, USA). ACPA, *N*-(piperidin-1-yl)-5-(4-iodophenyl)-1-(2,4-dichlorophenyl)-4-methyl-1H-pyrazole-3-carboxamide (AM 281), 1-[6-[[(17*b*)-3-methoxyestra-1,3,4(10)-trien-17-yl]amino]hexyl]-1*H*-pyrrole-2,5-dione (U-73122), 1H-[1,2,4]oxadiazolo[4,3-a]quinoxalin-1-one (ODQ) and VDM 11 were purchased from Tocris Cookson (Ellisville, MO, USA). 1,6-Bis-(cyclohexyloximinocarbonylamino)-hexane (RHC-80267) was purchased from Biomol (Plymouth Meeting, PA, USA). All other drugs, including 4-diphenylacetoxy-*N*-methylpiperidine methiodide, Rp-β-phenyl-1,N2-etheno-8-bromoguanosine 3′,5′-cyclic monophosphorothioate (Rp-8-Br-PET-cGMPS), diethylamine/NO complex and N_ω_-nitro-l-arginine methyl ester (L-NAME), were purchased from Sigma-Aldrich (St Louis, MO, USA).

### Immunofluorescence

Muscles were fixed in 3% paraformaldehyde for 1 h at 4 °C, rinsed for 1 h in physiological saline, permeablized for 30 min at 37 °C in 0.3% Triton X-100, preincubated for 15 min at room temperature (22–24 °C) in blocking solution (0.01% Triton X-100, 1% bovine serum albumin) and incubated in primary antibody (10 µg/mL of rabbit anti-human CB_1_ IgG no. 1; Alpha Diagnostic International, San Antonio, TX, USA) for 4 h at room temperature and then 12 h at 4 °C. Muscles were rinsed for 1 h in blocking solution, incubated with fluorescein-conjugated goat anti-rabbit IgG secondary antibody (5 µg/mL; American Qualex, San Clemente, CA, USA) for 2 h at 37 °C, rinsed in blocking solution for 30 min and mounted on slides with 20% glycerol in Slowfade Antifade® solution (Sigma-Aldrich). The antigen used to create the primary antibody was a 14-amino-acid peptide, referred to as CB11-P, which is found near the extracellular N-terminus of human CB_1_ (CB11-A; Alpha Diagnostic International). No punctate staining was observed when the secondary antibody was applied without the primary antibody.

To visualize the perisynaptic Schwann cells (PSCs), preparations were incubated for 15 min at room temperature with 1 µm POPO®-3 iodide nucleic acid stain (Molecular Probes) following the wash of the secondary antibody and then washed for 30 min in blocking solution. To visualize nerve terminals, the ends of cut nerve axons were loaded with Texas red dextran (Molecular Probes; 3000 MW, made in 10 mm HEPES, pH 7.2). Immediately following isolation of the *Ceratomandibularis* muscle and its associated nerve, the cut end of the nerve axon was placed into a small (1–2 µL) well containing 20 mm Texas red dextran. The Texas red dextran was allowed to load through anterograde transport at 9 °C for 16–18 h and then at 4 °C for an additional 2–3 h. After the nerve terminals had been filled with Texas red dextran, the tissue was processed for immunofluorescence as described above.

After being stained, NMJs were observed with a laser scanning confocal microscope manufactured by Prairie Technologies (Middleton, WI, USA) connected to a Nikon inverted microscope with a 60× oil immersion objective (1.4 numerical aperture). Images were manipulated and displayed using metamorph® software (v6.3, Universal Imaging, Downingtown, PA, USA).

### Electrophysiology

**Fig. 2:**
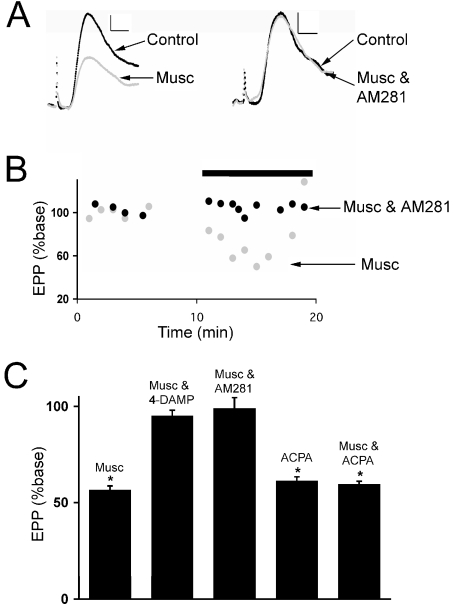
M_3_-mediated synaptic inhibition requires activation of CB_1_ receptors. (A) Representative end-plate potentials (EPPs) recorded before and after the application of muscarine (Musc). *N*-(piperidin-1-yl)-5-(4-iodophenyl)-1-(2,4-dichlorophenyl)-4-methyl-1H-pyrazole-3-carboxamide (AM 281) (5 µm) was present along with muscarine (5 µm) while recording the EPP traces shown on the right. Resting membrane potentials were approximately −90 mV. Calibration bars, 0.5 mV, 2 ms. (B) Time course of EPP amplitudes from two representative experiments plotted relative to the initial baseline amplitudes. The application of either 5 µm muscarine alone (grey) or along with 5 µm AM 281 (black) is indicated by the horizontal bar. Each data point represents the mean amplitude of eight sweeps. (C) Mean EPP amplitude (presented as percent of baseline) after 2–10-min applications of muscarine (5 µm) or arachidonylcyclopropylamide (ACPA) (10 µm). Muscarine was applied either alone (*n* = 4), with the M_3_ antagonist 4-diphenylacetoxy-*N*-methylpiperidine methiodide (4-DAMP) (10 µm, *n* = 4) or with the CB_1_ receptor antagonist AM 281 (5 µm, *n* = 4). ACPA was applied alone (*n* = 11) or with muscarine (5 µm, *n* = 4). *The mean EPP amplitude is significantly different from control (*P* < 0.05; Student's *t*-test). Error bars represent SEM.

**Fig. 3:**
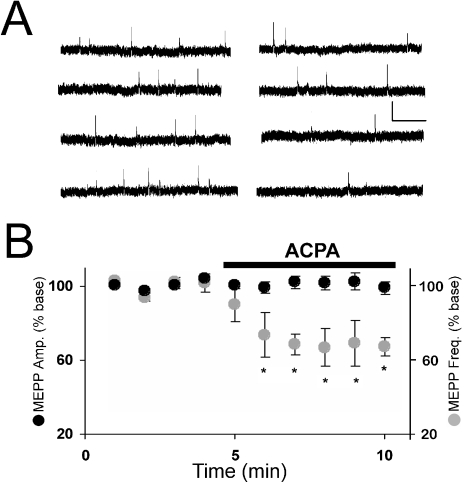
The cannabinoid agonist arachidonylcyclopropylamide (ACPA) does not change the amplitude of miniature end-plate potentials (MEPPs) but decreases their frequency. (A) Sample MEPPs recorded before (left) and after (right) the application of ACPA (10 µm). Calibration bars, 0.1 mV, 0.5 s. (B) Data points represent either the mean amplitude (black) or the mean frequency (grey) of MEPPs recorded during 1-min intervals before and during the application of ACPA (5 µm), indicated by the black bar. All data are expressed as a percent of the mean amplitude or frequency before application of ACPA (i.e. % baseline). Initial MEPP amplitudes and frequencies ranged from 0.5 to 1.5 mV and 0.1–1.0 Hz, respectively. Resting membrane potentials were at least −80 mV. *Mean is significantly different from baseline (*P* < 0.05; Student's *t*-test).

**Fig. 5:**
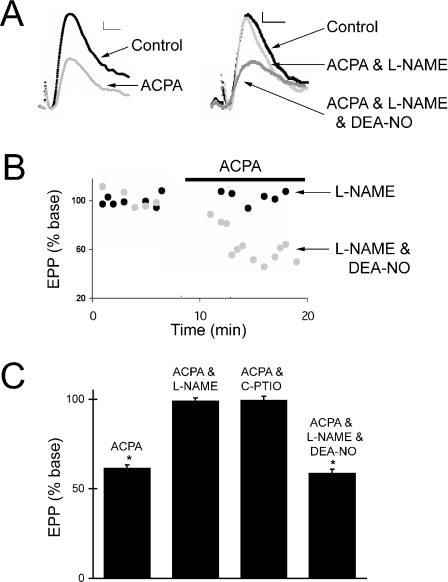
Arachidonylcyclopropylamide (ACPA)-induced synaptic inhibition requires nitric oxide. (A) Representative end-plate potentials (EPPs) recorded before and after the application of ACPA (10 µm). Either N_ω_-nitro-l-arginine methyl ester (L-NAME) or L-NAME and diethylamine/NO complex (DEA-NO) (traces on the right) were present during the recording of the EPPs. Each trace represents the average of eight sweeps. Resting membrane potentials were approximately −90 mV. Calibration bars, 0.5 mV, 2 ms. (B) Time course of EPP amplitudes from two representative experiments. The application of ACPA (10 µm) to a neuromuscular junction (NMJ) exposed to either L-NAME (black) or L-NAME and DEA-NO (grey) is indicated by the horizontal bar. Each data point represents the amplitude of an average of eight AC-coupled sweeps. (C) Mean percent reduction of EPP amplitudes (from initial baseline readings) following 5–10 min of ACPA (10 µm) application. ACPA was applied either alone (*n* = 11), with L-NAME (0.3 mm, *n* = 5), with 2-(4-carboxyphenyl)-4,4,5,5-tetramethylimidazoline-1-oxyl-3-oxide potassium salt (C-PTIO) (40 µm, *n* = 4) or with L-NAME and DEA-NO (0.1 mm, *n* = 5). *The mean EPP amplitude is significantly different from when it was measured under baseline conditions (*P* < 0.05; Student's *t*-test). Baseline measurements were made either under control conditions, in the presence of L-NAME, in the presence of C-PTIO or in the presence of L-NAME and DEA-NO, as appropriate for the experiment. Error bars represent SEM.

**Fig. 6:**
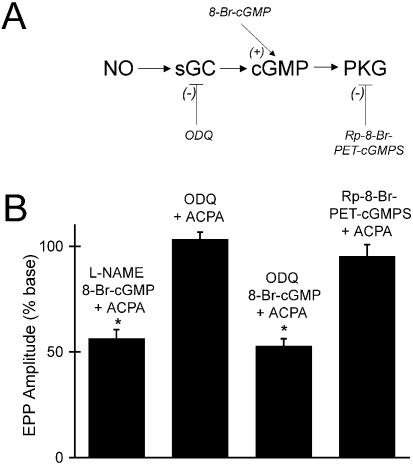
Nitric oxide (NO) is required to increase cGMP synthesis and activate protein kinase G. (A) The NO-cGMP signalling pathway. NO-stimulated soluble guanylyl cyclase (sGC) and cGMP-dependent protein kinase-1 (PKG). 1H-[1,2,4]oxadiazolo[4,3-a]quinoxalin-1-one (ODQ) is an inhibitor of sGC, Rp-B-Phenyl-1, N^2^-etheno-8-bromoguanosine 3’,5’-cyclic monophosphorothioate (Rp-8-Br-PET-cGMPS) is an inhibitor of PKG and 8-Br-cGMP is a membrane-permeable analogue of cGMP. (B) Mean percent reduction of end-plate potential (EPP) amplitudes following application of arachidonylcyclopropylamide (ACPA) (10 µm). ACPA was applied with N_ω_-nitro-l-arginine methyl ester (L-NAME) (0.3 mm) and 8-Br-cGMP (40 µm, *n* = 6) with ODQ (50 µm, *n* = 4), ODQ and 8-Br-cGMP (*n* = 4) and Rp-8-Br-PET-cGMPS (30 µm, *n* = 4). *The mean EPP amplitude is significantly different from its measurement under baseline conditions (*P* < 0.05; Student's *t*-test). Baseline EPP measurements were made in the presence of L-NAME and 8-Br-cGMP, ODQ, ODQ and 8-Br-cGMP, or Rp-8-Br-PET-cGMPS, respectively. Error bars represent SEM.

**Fig. 7:**
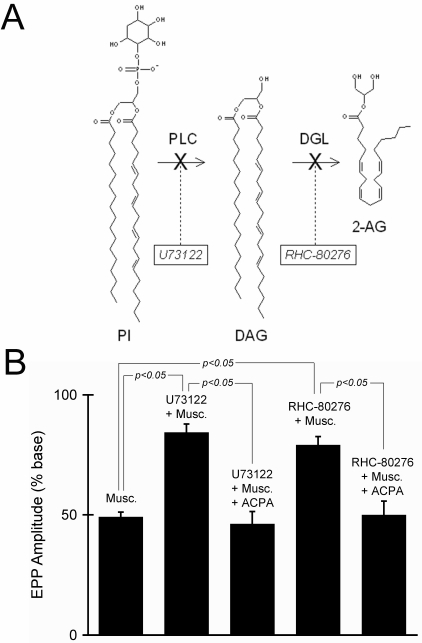
Endocannabinoids are produced through a phospholipase C (PLC)- and 1,2-diacylglycerol (DAG)-mediated pathway. (A) Biosynthetic pathway for 2-arachidonoylglycerol (2-AG). PI, phosphatidylinositol; DGL, diacylglycerol lipase. (B) Mean percent reduction of end-plate potential (EPP) amplitude following application of muscarine (Musc.) (5 µm). Muscarine was applied alone (*n* = 5), with the PLC inhibitor 1-[6-[[(17*b*)-3-methoxyestra-1,3,4(10)-trien-17-yl]amino]hexyl]-1*H*-pyrrole-2,5-dione (U-73122) (5 µm, *n* = 12), with U-73122 and arachidonylcyclopropylamide (ACPA) (*n* = 4), with the DGL inhibitor 1,6-bis-(cyclohexyloximinocarbonylamino)-hexane (RHC-80267) (200 µm, *n* = 5), and with RHC-80267 and ACPA (*n* = 3). All of the means were significantly different from baseline measurements made under control conditions (*P* < 0.05; Student's *t*-test). The Student's *t*-test was also used to calculate the statistical significance of the differences between each individual mean. None of the differences were significant except for those indicated on the graph. Error bars represent SEM.

**Fig. 10:**
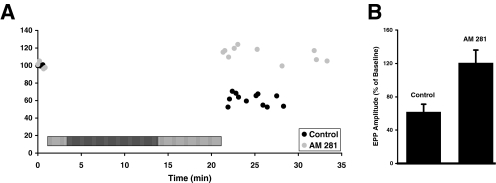
Synaptic depression requires functional CB_1_ receptors. (A) Time course of end-plate potential (EPP) amplitudes from two representative experiments in which the motor nerve was stimulated for 20 min at 1 Hz (indicated by the horizontal hatched bar). The experiment was performed either under control conditions (black) or in the presence of *N*-(piperidin-1-yl)-5-(4-iodophenyl)-1-(2,4-dichlorophenyl)-4-methyl-1H-pyrazole-3-carboxamide (AM 281) (grey). Each data point represents the amplitude of an average of eight sweeps. (B) Mean percent reduction of EPP amplitudes (from initial baseline readings) following 20 min of continuous 1-Hz stimulation of the motor nerve. Stimulation was delivered either under control conditions (*n* = 11) or in the presence of AM 281 (1 µm; *n* = 7). The mean EPP amplitudes under these two conditions are significantly different from each other (*P* < 0.05; Student's *t*-test). Baseline measurements were either made under control conditions or in the presence of AM 281, as appropriate for the experiment. Error bars represent SEM.

End-plate potentials were evoked by stimulating the motor nerve axon with a continuous train of depolarizing square pulses of 1–10 V, 0.04 ms duration, at 0.25 Hz (or, for the conditioning stimuli used in Fig. 10, 1 Hz). EPPs were measured using glass micropipettes filled with 3 m KCl (20–40 MΩ). Membrane potentials were amplified with a Cell Explorer (Dagan Instruments, Minneapolis, MN, USA) and collected with a MacLab data acquisition system (AD Instruments, Colorado Springs, CO, USA). For the experiments depicted in Figs 2, 5, 6, 7 and 10, EPPs were recorded from randomly selected muscle fibers. Each trial (*n*) represents the mean EPP amplitude recorded at five to eight locations (i.e. NMJs) in a single preparation. The electrode was inserted in each muscle cell only long enough to record between four and 16 EPPs that were filtered to reject direct current (i.e. 16–64 s), which were averaged online and the maximum amplitude measured offline. For the experiment depicted in Fig. 3, the intracellular recording electrode was inserted into a single muscle fibre and left in place long enough to record at least 100 miniature EPPs (MEPPs) before and during the application of ACPA (approx. 10 min). For the experiments depicted in Fig. 8, the intracellular electrode was filled with 1.5 m KCl (rather than 3 m KCl) and inserted into a muscle cell just long enough to record between four and eight EPPs (i.e. 8–32 s). The electrode was carefully retracted until it was just outside the muscle. The electrode was then reinserted at the same spot after waiting at least 1 min. This process was repeated up to 10 times. In these experiments, *n* refers to the number of muscle cells (i.e. NMJs). Student's *t*-test (two-sample assuming equal variance) was used to evaluate the significance of all electrophysiological data.

### Intracellular injection

VDM 11 (dissolved in Tocrisolve®) or Tocrisolve® itself was injected into muscle cells within 100 µm of the end plate through a glass micropipette with a tip diameter less than 0.1 µm. The electrode was filled with 7 µm VDM 11 (or the corresponding volume of Tocrisolve®) and rhodamine B to monitor the progress of the injection. Between 10 and 20 5-s pulses (30 p.s.i.) were applied via a pneumatic pico pump (PV 830; World Precision Instruments). When VDM 11 was applied directly to the bathing solution, its final concentration was at least 7 µm.

### Calcium imaging

**Fig. 4:**
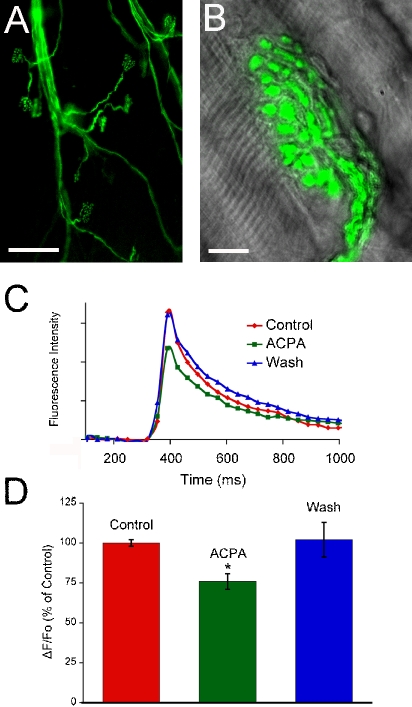
The CB_1_ receptor agonist arachidonylcyclopropylamide (ACPA) reduces the calcium transient in the motor nerve terminal triggered by a single nerve impulse. (A) Low magnification image of a preparation loaded with calcium green-1 dextran. The dye has been loaded into a few axons and made its way into the nerve terminals. Scale bar, 100 µm. (B) An overlay of the calcium green-1 dextran fluorescence emission onto an image collected using differential-interference contrast (Nomarski) optics. Both images were taken with a 60× water immersion objective (numerical aperture 1.0) and a Coolsnap camera (Photometrics). Scale bar, 10 µm. (C) Fluorescence emission intensity is plotted in arbitrary units as a function of time relative to the stimulus pulse used to elicit an action potential in the motor nerve. Three traces obtained during the perfusion with normal physiological saline (Control), following the application of 5 µm ACPA for 5 min (ACPA) and following the wash-out of ACPA with normal saline for 20 min (Wash). (D) Mean amplitudes of the calcium transients measured under the three conditions: Control (*n* = 12), ACPA (*n* = 12) and Wash (*n* = 9). The application of 10 µm ACPA results in a significant (*P < 0.05 Student's *t*-test) and reversible decrease in the calcium transients induced by a single action potential in the motor nerve.

Wide-field epifluorescence microscopy was used to measure calcium transients in motor nerve terminals loaded with the fluorescent Ca^2+^ indicator calcium green-1 dextran (Molecular Probes). Calcium green-1 dextran (3000 or 10 000 MW) was back-loaded into nerve terminals using the same technique described previously for loading Texas red dextran. The imaging was performed on a Nikon Eclipse (TE2000-E) inverted microscope with a 60× water immersion objective (numerical aperture 1.0) with an additional 1.5× magnification for a final magnification of 90×. A standard filter cube optimized for fluorescin isothiocyeuate (FITC) was used. The camera for the imaging experiments was a Cascade 512B cooled charge-coupled device (CCD) camera (Photometrics, Tucson, AZ, USA) that utilizes impact-ionization for low-noise signal gain. Images were acquired as a time lapse (50 or 100 10-ms exposures with a delay of 25 ms between exposures due to the internal memory transfer and buffering of the camera) and then compiled into a stack (metamorph v6.3). After collecting five to six images, which were used to establish baseline fluorescence, a stimulus (a suprathreshold 1–10-V square pulse 0.04 ms in duration) was delivered to the nerve. The stimulus was synchronized with the image capture by using trigger pulses (generated by metamorph v6.3) and two Macintosh-driven PowerLab instruments (400 and 4SP, AD Instruments). The speed of the image acquisition and proper synchronization allowed for the recording of the complete time-course of the calcium signal. The data were analysed by selecting a single bouton as a region of interest and measuring the average light intensity. After subtracting the average light intensity from a region not associated with the nerve terminal (i.e. background), the average light intensity was plotted as a function of time (see Fig. 4C).

## Results

### CB_1_ receptors are concentrated on the presynaptic nerve terminal

**Fig. 1:**
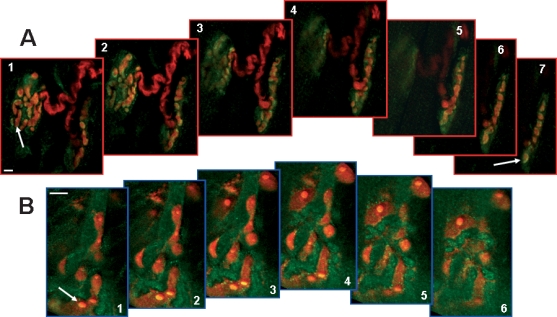
Immunofluorescence localization of CB_1_ receptors at the neuromuscular junction (NMJ). (A) Distribution of CB_1_ receptors is shown in two NMJs in which the motor nerve terminal is costained with Texas red dextran. Most of the CB_1_ receptors (green) are seen as yellow as they overlay the nerve terminal (red). The tissue was prepared as described in Materials and methods and imaged using a Prairie confocal microscope set to collect 25 image planes at 0.5-µm intervals. Every fourth confocal image is shown proceeding from the top of the collected stack to the bottom (i.e. each image is 2 µm below or above the adjacent image). Arrows point to areas in which the CB_1_ receptors are observed around the periphery of presynaptic boutons. (B) CB_1_ receptors are shown in an NMJ in which the perisynaptic Schwann cells (PSCs) are costained with POPO®-3. The relative lack of overlap between the CB_1_ receptors (green) and PSCs (red) suggests that PSCs express fewer CB_1_ receptors than does the nerve. The tissue was prepared as described in Materials and methods and imaged using a Prairie confocal microscope set to collect six image planes at 0.5-µm intervals. The confocal images shown proceed from top to bottom. The arrow points to a PSC that appears to express CB_1_ receptor. Scale bars, 10 µm.

Most of the biological effects of cannabinoids are mediated through specific membrane receptors. Of the two subtypes that have been discovered and cloned, the CB_1_ receptor exists primarily in the nervous system with the CB_2_ receptor located mainly in immune tissue (Howlett *et al.*, 2002; but see Van Sickle *et al.*, 2005). We used immunofluorescence to determine whether the CB_1_ receptor is present at the lizard NMJ. As seen in Fig. 1, we detected considerable staining with antibodies to the CB_1_ receptor. To determine specifically where the receptors are located at the NMJ, we back-filled the nerve terminals with Texas red dextran (see Materials and methods) and processed the tissue for immunofluorescence, using fluorescein-labelled secondary antibodies to reveal the CB_1_ receptors. The results, shown in Fig. 1A, indicate that CB_1_ receptors are found mostly on the nerve terminals. We did observe some CB_1_ receptor staining that did not colocalize with the nerve terminal and therefore appears green but most was clearly on the nerve terminal and therefore appears yellow because it overlays Texas red dextran. Careful examination of 0.5-µm confocal planes revealed that most of the CB_1_ receptors were located along the periphery of the nerve terminal branches and boutons, presumably associated with the cell membrane (see arrows in Fig. 1A). As a control, two preparations were exposed to the secondary antibody without the primary anti-CB_1_ antibody. No fluorescence could be detected in these preparations.

To further establish the localization of the CB_1_ receptors, PSCs, glial cells that closely envelope the nerve terminals, were stained using the nucleic acid stain POPO®-3 iodide (Molecular Probes). As the nerve terminals do not contain nucleic acids, POPO®-3 uniquely identifies the PSCs. The tissue was also processed for immunofluorescence, using fluorescein-labelled secondary antibodies to locate the CB_1_ receptors. Using confocal microscopy, we determined that a small amount of CB_1_ receptors are present on the PSCs. Figure 1B shows six confocal images collected at 0.5-µm intervals. Although there is a small amount of overlap between the green (CB_1_ receptor) and red (PSCs) signals, a close examination of the individual confocal images reveals that most of the CB_1_ receptors do not overlap with the PSCs but are located above, below or between the PSCs. Our observations of 12 preparations costained with CB_1_ antibodies and either POPO®-3 or Texas red dextran (to identify PSCs or nerve terminals, respectively) allow us to conclude that the CB_1_ receptors are concentrated in cell membranes of the motor nerve terminals at the lizard NMJ. However, our observations do not allow us to exclude the possibility that some CB_1_ receptors are expressed on the PSCs. We did not observe any CB_1_ immunofluorescence associated with the muscle cells (data not shown).

### M_3_ and CB_1_ antagonists block muscarine-induced synaptic depression

In previous work muscarine has been shown to modulate synaptic transmission at the lizard NMJ in a biphasic manner, first depressing and then enhancing neurotransmitter release (Graves *et al.*, 2004). In addition to being temporally separable, the depression and enhancement are also pharmacologically distinct; the M_3_ mAChR subtype mediates the depression and the M_1_ subtype mediates the enhancement (Graves *et al.*, 2004). To determine whether the CB_1_ receptor plays a role in either (or both) phase(s), the CB_1_ antagonist AM 281 was applied. Although AM 281 had no effect when applied by itself (data not shown), when muscarine was applied in the presence of AM 281 the first phase (depression) was precluded whereas the second phase (enhancement) was unaffected. Thus, we focused the remainder of our investigation on the depression of neurotransmission triggered by muscarine.

Figure 2 depicts the unique pharmacological sensitivity of the first phase of muscarine's influence on synaptic transmission. Following 5–10 min of muscarine application, the EPP amplitude was reduced by 43.4 ± 2.1% (mean ± SEM), which is significantly different from baseline measurements taken prior to muscarine application (*P* < 0.05; Fig. 2A, left and C). The M_3_ receptor antagonist 4-diphenylacetoxy-*N*-methylpiperidine methiodide prevents this. In the presence of 4-diphenylacetoxy-*N*-methylpiperidine methiodide, muscarine reduced the EPP amplitude by only 4.9 ± 3.0%, a change not significantly different from baseline (Fig. 2C). In a similar manner, in the presence of the CB_1_ receptor antagonist AM 281, the EPP amplitude decreased by only 1.0 ± 5.7%, which is also not significantly different from control (see Fig. 2A, right, B and C). Thus, the depression of synaptic transmission at the lizard NMJ by muscarine requires functional M_3_ and CB_1_ receptors.

The ability of the CB_1_ antagonist AM 281 to block muscarine-induced synaptic depression suggests that eCBs mediate this effect. To test this suggestion, the CB_1_ agonist ACPA was applied. ACPA reduced the EPP amplitude by approximately the same amount as muscarine (39.1 ± 3.2 vs. 43.4 ± 2.1%; Fig. 2C). Furthermore, ACPA reduces the EPP amplitude over approximately the same time course as muscarine (compare Fig. 2B with 5B). After 5–10 min exposure to ACPA, the EPP amplitude was significantly different from baseline (*P* < 0.05) but not significantly different from the EPP amplitude after 5–10 min exposure to muscarine.

To provide further evidence that an eCB mediates the muscarine-induced depression, preparations were exposed to both muscarine and ACPA. The EPP amplitude was reduced by a mean of 40.4 ± 1.5% after 5–10 min exposure to 5 µm muscarine and 10 µm ACPA. The mean was significantly different from baseline measurements (*P* < 0.05) but not different from the EPP amplitude in the presence of either muscarine or ACPA alone. The ability of ACPA to occlude the effect of muscarine is consistent with muscarine acting via the release of an eCB that subsequently inhibits synaptic transmission by activating a presynaptic CB_1_ receptor.

### Cannabinoid-induced synaptic depression is presynaptic

The synaptic depression induced by muscarine has been shown previously to be of presynaptic origin; the activation of M_3_ receptors at the lizard NMJ reduces the evoked release of neurotransmitter (Graves *et al.*, 2004). To see if the same is true of the cannabinoid agonist ACPA, spontaneous MEPPs were recorded in three preparations before and during the application of ACPA. As shown in Fig. 3, the mean amplitude of MEPPs was unchanged, indicating that the reduction of the EPP amplitude induced by ACPA (Fig. 2C) must be due to a decrease in the quantal content of evoked neurotransmitter release (Del Castillo & Katz, 1954). Although ACPA had no effect on the MEPP amplitude, it did significantly reduce the frequency of MEPPs (Fig. 3B), which is also consistent with a presynaptic action. We also note that the time-course of individual MEPPs was unchanged by ACPA.

### Cannabinoids decrease the evoked calcium transient in nerve terminal

At synapses where it has been possible to make the necessary measurements, the activation of CB_1_ receptors has been shown to reduce the stimulus-induced calcium transient in the presynaptic nerve terminal (Twitchell *et al.*, 1997; Sullivan, 1999; Schweitzer, 2000; Kreitzer & Regehr, 2001; Robbe *et al.*, 2001; Brown *et al.*, 2004; Daniel *et al.*, 2004; Kushmerick *et al.*, 2004). However, there is also evidence in some cells that cannabinoids inhibit a step downstream of Ca^2+^ entry (Takahashi & Linden, 2000; Vaughan *et al.*, 2000). To determine how eCBs inhibit neurotransmitter release at the lizard NMJ, we measured calcium transients in motor terminals during stimulus-evoked action potentials. The fluorescent calcium indicator calcium green-1 (conjugated to dextran, 3000 or 10 000 MW) was loaded specifically into motor nerve terminals via anterograde axoplasmic transport (Fig. 4A). If the dye was prevented from leaking out of the well directly into the physiological saline with a Vaseline® seal, calcium green-1 dextran was observed exclusively in the nerve terminals (see Fig. 4B).

Presynaptic calcium transients were measured by delivering a single supra-threshold stimulus to the motor axon while imaging a motor nerve loaded with calcium green-1 dextran. A typical series of calcium transients is plotted in Fig. 4C, showing the change in calcium concentration (as fluorescence emission intensity) before, during and after the application of the CB_1_ agonist ACPA. The peaks of the transients were measured and plotted for each condition (Fig. 4D). Compared with measurements made in control (i.e. normal) saline, ACPA (10 µm) reduced the peak calcium concentration by 24.1 ± 4.9%. The mean calcium peak measured in the presence of ACPA was significantly different (*P* < 0.05) from the mean calcium peaks measured both before applying ACPA and after washing with normal saline.

To determine whether a 24% decrease in the peak Ca^2+^ concentration is sufficient to decrease neurotransmitter release by the amount observed when CB_1_ receptors are activated by ACPA (∼40%, Fig. 2C), we carried out the following experiment. Using calcium green-1-loaded nerve terminals we determined that we could lower the evoked calcium transient in the motor nerve terminals by 25 ± 4% (*n* = 3) by reducing the concentration of Ca^2+^ in the external physiological saline to 1.2 mm (from 2.0 mm), i.e. by reducing the external Ca^2+^ we could reduce the peak of the calcium transient by the same amount observed when ACPA is applied (in a solution with a normal Ca^2+^ concentration). We then asked whether such a decrease was sufficient by itself to reduce EPPs by at least 40%. When we reduced the external Ca^2+^ concentration to 1.2 mm while measuring evoked EPPs, the EPP amplitudes dropped by 74 ± 4% (data not shown). Thus, the decrease in the size of the evoked calcium transient upon application of ACPA is more than sufficient to account for the observed inhibition of neurotransmitter release.

### Endocannabinoid-induced synaptic depression requires nitric oxide

As shown previously, the muscarine-induced depression of ACh release requires NO (Graves *et al.*, 2004). To see if ACPA has a similar requirement, muscles were pretreated with the NO synthase inhibitor L-NAME or the extracellular NO chelator carboxy-PTIO. Neither L-NAME nor carboxy-PTIO by themselves had an effect on the EPP amplitude (data not shown). However, when applied in the presence of L-NAME or carboxy-PTIO, ACPA did not significantly alter the EPP amplitude (a decrease of 1.2 ± 1.9 and 0.7 ± 2.5%, respectively; Fig. 5). This result suggests that ACPA does indeed require NO synthesis and diffusion through the extracellular space to depress synaptic transmission. However, it is also possible that L-NAME or carboxy-PTIO interfered with ACPA through a mechanism not involving NO synthesis or diffusion. To rule out the former possibility, ACPA was applied along with the NO donor diethylamine/NO complex to a preparation that had been pretreated with L-NAME. As seen in Fig. 5, diethylamine/NO complex restores ACPA's ability to inhibit synaptic transmission, reducing the EPP amplitude by 41.4 ± 2.3% (*P* < 0.05).

### Nitric oxide acts via soluble guanylate cyclase and protein kinase G

To gain further insight into the role of NO in the eCB-mediated inhibition of neurotransmitter release, we determined whether a membrane-permeable analogue of cGMP (8-Br-cGMP) could restore ACPA's ability to reduce the EPP amplitude in a preparation pretreated with L-NAME. As seen in Fig. 6 (compare with Fig. 5), 8-Br-cGMP was just as effective as diethylamine/NO complex in overcoming the block of NO synthase by L-NAME, reducing the EPP amplitude by 43.7 ± 4.3% (*P* < 0.05). This suggests that NO mediates its permissive effect by activating soluble guanylate cyclase. To test this idea further, the soluble guanylate cyclase inhibitor ODQ was applied 15 min before adding ACPA. In the presence of ODQ, ACPA did not have a significant effect on the EPP amplitude (an increase of 3.3 ± 3.4%). To verify the specificity of ODQ's effect, 8-Br-cGMP was shown to reconstitute ACPA's inhibition of synaptic transmission in the presence of ODQ (a decrease of 47.3 ± 3.5%, *P* < 0.05), presumably by bypassing the blocked soluble guanylate cyclase enzyme. Lastly, we tested whether cGMP-dependent protein kinase was necessary. As shown in Fig. 6, Rp-B-Phenyl-1, N^2^-etheno-8-bromoguanosine 3’,5’-cyclic monophosphorothioate (Rp-8-Br-PET-cGMPS), an inhibitor of cGMP-dependent protein kinase, also prevented ACPA from altering synaptic transmission (the EPP amplitude was decreased by 4.8 ± 5.5%).

### Phospholipase C and diacylglycerol lipase are required for the muscarine-induced depression of synaptic transmission

The two most well-studied eCBs, anandamide and 2-AG, are known to be synthesized from phospholipid precursors in the cell membrane (Freund *et al.*, 2003). Two main routes of synthesis have been postulated for 2-AG, one of which involves the ubiquitous enzyme phospholipase C (PLC) and diacylglycerol lipase (DGL) (Stella *et al.*, 1997). To ascertain whether 2-AG is involved in the muscarine-induced depression of ACh release, muscarine was applied to 12 nerve–muscle preparations that had been preincubated for at least 1 h with the PLC inhibitor U-73122. Under these conditions, muscarine reduced the EPP amplitude by a mean of only 15.7 ± 3.6%, which is significantly different from the decrease caused by muscarine alone (*P* < 0.01; Fig. 7B). However, when the CB_1_ agonist ACPA was applied to the same preparations that had been pretreated with U-73122, the EPP amplitude was decreased by an amount (53.8 ± 5.2%) that is indistinguishable from the effects of either ACPA or muscarine in the absence of U-73122 (compare Fig. 7B with Fig. 2).

As the synthesis of 2-AG via the pathway shown in Fig. 7A requires the enzyme DGL in addition to PLC, we also examined the effects of the DGL inhibitor RHC-80267. In five preparations preincubated for 1 h with RHC-80267, muscarine reduced the EPP amplitude by a mean of 20.9 ± 3.6%, which is also significantly different from the decrease caused by muscarine alone (Fig. 7B; *P* < 0.01). The likelihood that the action of RHC-80267 was due specifically to its inhibition of DGL was demonstrated by applying 10 µm ACPA to preparations that had been pretreated with RHC-80267 (and muscarine) and showing that ACPA still inhibited the EPP amplitude by its normal amount (i.e. 50.1 ± 5.9%).

Although the comparisons described above indicate that the enzymes PLC and DGL are responsible for a statistically significant component of the muscarine-inducued depression, it is noteworthy that muscarine still significantly reduced (*P* < 0.05) the EPP amplitude in the presence of either U-73122 or RHC-80267 (see Fig. 7).

### Endocannabinoids are released from the muscle via an endocannabinoid membrane transporter

Evidence has recently been presented that eCBs are released from striatal neurones in the rat brain by a membrane transporter that acts via facilitated diffusion (Ronesi *et al.*, 2004). To determine whether the same mechanism might be responsible for eCB release at the lizard NMJ and to establish the cellular source of the eCBs, we injected individual muscle cells with VDM 11, an inhibitor of eCB-facilitated diffusion (De Petrocellis *et al.*, 2000). Shortly after injecting VDM 11, EPPs were recorded from the same muscle fibre before and after the local application of muscarine. The average EPP amplitude was not significantly changed under these conditions (Fig. 8). In contrast, when muscles were injected with Tocrisolve®, the solvent in which VDM 11 was dissolved, or if VDM 11 was added to the bathing solution, muscarine significantly inhibited the EPP amplitude (by 58.1 ± 1.2 and 49.6 ± 8.2%, respectively; Fig. 8). In contrast to muscarine, when the CB_1_ receptor agonist ACPA was applied to muscle cells injected with VDM 11, the EPP amplitude was significantly depressed (45.8 ± 8.9%; Fig. 8). The only condition under which the EPP amplitude was not significantly inhibited was when muscarine was applied to muscles that had been injected with VDM 11. The small reduction of the EPP amplitude (10.9 ± 3.7%) was significantly different from each of the other conditions (*P* < 0.01; Fig. 8). These results collectively support the suggestion that eCBs are released from the postsynaptic muscle cells via facilitated diffusion (Ronesi *et al.*, 2004). These results also indicate that the muscle is the source of eCBs released at the vertebrate NMJ following the activation of M_3_ mAChRs.

### Endocannabinoids are involved in synaptic depression

The preceding experiments point to an essential role for eCBs in the inhibition of synaptic transmission following the activation of M_3_ mAChRs. To determine the physiological context under which eCBs might become deployed, we looked for an effect of the CB_1_ receptor antagonist AM 281 on a form of synaptic depression that most closely resembles the magnitude and time-course of the inhibition induced by either muscarine or ACPA. The results are shown in Fig. 10. Continuous stimulation of the motor nerve at 1 Hz for 20 min depresses the EPP amplitude by 38.2 ± 9.2%, a reduction that is not significantly different from that produced by either muscarine or ACPA (see Fig. 2). In the presence of AM 281, however, stimulation of the motor nerve (20 min, 1 Hz) failed to depress synaptic transmission, resulting in a mean EPP amplitude that is 120.7 ± 15.3% of baseline, an amplitude that is significantly different from that produced in the absence of AM 281 (*P* < 0.05; Fig. 10).

## Discussion

To our knowledge, this is the first report of cannabinoid receptors at a vertebrate striated NMJ. Although the CB_1_ receptor has been identified throughout the central and peripheral nervous systems in several species (see Howlett *et al.*, 2002) and its effects have been detected at the frog NMJ (Turkanis & Karler, 1986; Van der Kloot, 1994), the receptor has never been localized specifically at the NMJ of any species. Our observation that CB_1_ receptors are concentrated on the motor nerve terminals is consistent with its preferential expression on presynaptic nerve terminals elsewhere in the nervous system (see Kreitzer & Regehr, 2002; Wilson & Nicoll, 2002). However, our immunofluorescence studies also suggest that CB_1_ receptors may be on the closely associated PSCs, albeit at a lower density (Fig. 1B). The significance of this observation has yet to be explored.

**Fig. 9:**
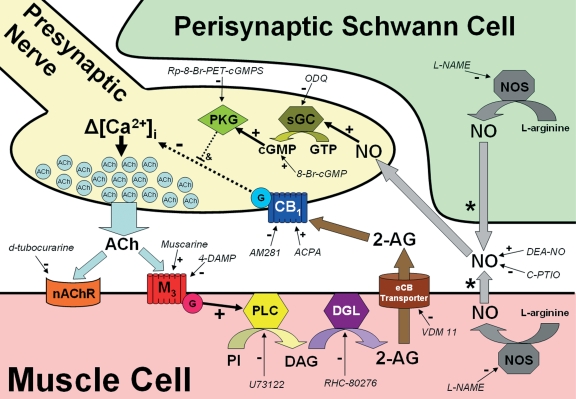
Proposed model summarizing the role played by endocannabinoids (eCBs) at the vertebrate neuromuscular junction. This represents our current working model for explaining the signalling pathways involved in muscarine-induced synaptic depression at the vertebrate neuromuscular junction. Block arrows represent the diffusion or movement of a signalling molecule. Curved block arrows indicate an enzymatic conversion. Solid black arrows depict steps that have been experimentally verified, whereas dashed arrows reveal steps that contain unknown details. All chemicals in italics and their respective arrows are meant to show the various targets of each of the experimental reagents used (see text for details). We are not sure whether nitric oxide (NO) is produced in the muscle fibers or the perisynaptic Schwann cells so we have included each possibility and noted both with an asterisk. NO, acting via cGMP-dependent protein kinase (PKG), is necessary but not sufficient to modulate neurotransmitter release and we have noted this with a dashed line and & symbol. We do not yet know the specific target of PKG. Δ[Ca^2+^]_i_, intracellular calcium transient; ACh, acetylcholine; ACPA, arachidonylcyclopropylamide; 2-AG, 2-arachidonylglycerol; AM 281, *N*-(piperidin-1-yl)-5-(4-iodophenyl)-1-(2,4-dichlorophenyl)-4-methyl-1H-pyrazole-3-carboxamide; CB_1_, cannabinoid receptor subtype 1; cGMP, cyclic guanosine monophosphate; DAG, diacylglycerol; 4-DAMP, 4-diphenylacetoxy-*N*-methylpiperidine methiodide; DGL, diacylglycerol lipase; G, G-protein; GTP, guanosine triphosphate; L-NAME, N_ω_-nitro-l-arginine methyl ester; M_3_, muscarinic acetylcholine receptor subtype 3; nAChR, nicotinic acetylcholine receptor; NOS, NO synthase; ODQ, 1H-[1,2,4]oxadiazolo[4,3-a]quinoxalin-1-one; PI, phosphatidylinositol or its phosphorylated derivatives; PLC, phospholipase C; RHC-80267, 1,6-bis-(cyclohexyloximinocarbonylamino)-hexane; Rp-8-Br-PET-cGMPS; sGC, soluble guanylate cyclase; U-73122, 1-[6-[[(17b)-3-methoxyestra-1,3,4(10)-trien-17-yl]amino]hexyl]-1H-pyrrole-2,5-dione; VDM 11, (5Z,8Z,11Z,14Z)-*N*-(4-hydroxy-2-methylphenyl)-5,8,11,14-eicosatetraenamide.

We carried out this investigation at the NMJ of the lizard because previous work had shown that: (i) activation of M_3_ mAChRs depresses neurotransmitter release at this synapse (Graves *et al.*, 2004) and (ii) eCBs mediate a similar suppression of neurotransmitter release induced by M_3_ receptor activation in the hippocampus (Fukudome *et al.*, 2004). The results reported here suggest that eCBs play a similar role at these two synapses. The model presented in Fig. 9 summarizes the role of eCBs at the vertebrate NMJ suggested by the experiments described in this work. Activation of M_3_ receptors on the muscle cell triggers the synthesis of eCBs, probably 2-AG, that are released via a transporter in the muscle membrane. Once in the synaptic cleft, 2-AG binds to CB_1_ receptors on the presynaptic nerve terminal, reduces the action-potential-induced Ca^2+^ transient and thereby reduces neurotransmitter release. NO, produced in either the muscle or PSCs, is required for one or more of the steps depicted in Fig. 9.

### Mechanism of action of endocannabinoids

Previous studies are consistent with our conclusion that eCBs suppress synaptic transmission presynaptically (Kreitzer & Regehr, 2002; Wilson & Nicoll, 2002) and by decreasing the calcium transient in the presynaptic nerve terminal. In some neurones, cannabinoids have been shown to inhibit presynaptic voltage-dependent Ca^2+^ channels (e.g. see Kushmerick *et al.*, 2004). In other neurones, cannabinoids have been found to activate presynaptic K^+^ channels (e.g. Schweitzer, 2000; Robbe *et al.*, 2001; Daniel *et al.*, 2004). In either case, cannabinoids reduce the depolarization-induced Ca^2+^ transient in the presynaptic terminal (see Kreitzer & Regehr, 2001) and thereby decrease the release of neurotransmitter. Our results are consistent with either possibility; future work will be necessary to elucidate the specific mechanism at the NMJ.

Our observation that the cannabinoid agonist ACPA reduces the frequency of MEPPs (see Fig. 3) has been reported by others (Takahashi & Linden, 2000; Vaughan *et al.*, 2000; Gerdeman & Lovinger, 2001). Such observations have been used to support a mechanism of action for cannabinoids that is downstream of Ca^2+^ influx, i.e. a direct action on the secretory machinery. This appears to conflict with most investigations, including the present one (Fig. 4C and D), which have reported that activation of CB_1_ receptors causes a significant reduction in presynaptic Ca^2+^ influx (Brown *et al.*, 2004; Twitchell *et al.*, 1997; Sullivan, 1999; Schweitzer, 2000; Kreitzer & Regehr, 2001; Robbe *et al.*, 2001; Daniel *et al.*, 2004; Kushmerick *et al.*, 2004). These apparent discrepancies may reflect different mechanisms of action for eCBs. Alternatively, they may reflect different degrees of coupling between calcium channels and the presynaptic vesicle release complex (e.g. see Spafford & Zamponi, 2003). We have not yet investigated this further; however, the vertebrate NMJ is ideal for answering such mechanistic questions and we eagerly anticipate using this preparation to clarify such questions related to eCB-mediated synaptic modulation.

### Requirement for nitric oxide

In addition to being the first description of a physiological role for eCBs at the vertebrate NMJ, this is also the first time that the mechanism of action of eCBs has been directly shown to depend on NO (see Fig. 5). There have been numerous reports suggesting a linkage between eCBs and NO (e.g. see Randall & Kendall, 1998; Waksman *et al.*, 1999; Azad *et al.*, 2001; Namiki *et al.*, 2005); however, to our knowledge, the cellular mechanism of action of eCBs in the nervous system has never been linked to an absolute requirement for NO. The dependence of eCBs on NO reported in this work may be unique to the NMJ. Alternatively, it may be a more general phenomenon that has not been considered elsewhere. Our results indicate that eCB-mediated synaptic modulation requires NO; however, as shown previously for muscarine-induced synaptic modulation (Graves *et al.*, 2004), NO is necessary but not sufficient. The observation that NO synthase is present in all three cellular components at the NMJ, the nerve terminal, muscle and PSC (Graves *et al.*, 2004), makes it difficult to determine the essential source of NO. However, we do know that NO must be synthesized in a different cell than its target as chelating extracellular NO with carboxy-PTIO abolishes synaptic modulation (Fig. 5). Therefore, we postulate that NO is synthesized in the muscle or PSC and diffuses to the nerve terminal where it activates soluble guanylate cyclase (see Fig. 9). Evidence obtained at the amphibian NMJ supports the suggestion that the muscle is the source of NO, which is generated either tonically (Thomas & Robitaille, 2001) or in response to indirect low-frequency stimulation (Etherington & Everett, 2004).

### Evidence that 2-arachidonoylglycerol is responsible for muscarine-induced depression

Our results implicate 2-AG as the eCB at the vertebrate NMJ (Fig. 7). It is worth noting that we were unable to completely abolish muscarine-induced synaptic depression by inhibiting PLC or DGL (compare Figs 2 and 7). This either means that the inhibitors that we used, U-73122 and RHC-80276, did not fully eliminate the activity of PLC and DGL or that there is another pathway for synthesizing eCBs at the lizard NMJ. In addition to using PLC and DGL, phosphatidylinositol can also be converted to 2-AG via phospholipase A1 and lyso-PLC (Freund *et al.*, 2003). Thus, the residual synaptic depression observed in Fig. 7 may have been due to the synthesis of 2-AG via this latter pathway (not shown). It is also possible that another cannabinoid, such as anandamide, is also released by the activation of M_3_ receptors at the NMJ. Additional experiments are needed to distinguish between these possibilities.

### Mechanism of endocannabinoid release at the vertebrate neuromuscular junction

Regardless of whether the eCB at the vertebrate NMJ is exclusively 2-AG or is 2-AG and anandamide, it is clear from this study that the eCB is synthesized in the muscle and is released by a membrane transporter (see Fig. 8). The idea of injecting VDM 11 in the muscle was inspired by Ronesi *et al.* (2004) who had shown that injection of VDM 11 into postsynaptic cells in corticostriatal brain slices from the rat abolished a long-term depression known to involve eCBs. As noted by Ronesi *et al.* (2004), the transporter previously shown to be responsible for the uptake of anandamide and 2-AG via the process of facilitated diffusion (Beltramo *et al.*, 1997; Hillard *et al.*, 1997) would also be able to transport either eCB out of the cell. The net direction of the movement would simply depend on the relative concentration of eCB across the membrane. Thus, in a cell such as a striatal neurone or a vertebrate muscle in which eCBs are rapidly synthesized, the transporter will function to release the eCBs into the surrounding synaptic gap. We believe that this is what is happening at the vertebrate NMJ.

### Physiological relevance of endocannabinoids at the vertebrate neuromuscular junction

Under physiological conditions the muscarinic receptors at the vertebrate NMJ are presumably activated by ACh released from the motor nerve terminal during the process of synaptic transmission. The results presented here suggest that 20 min of 1-Hz stimulation is sufficient to activate the release of eCBs, which then depress synaptic transmission by activating CB_1_ receptors (Fig. 10). The simplest explanation, and the one that is most consistent with the pharmacological results presented in this work, is that ACh activates M_3_ mAChRs, which then elicits the synthesis (Fig. 7) and release (Fig. 8) of eCBs. However, there are other possibilities. Glutamate, which has been shown to be involved in synaptic depression at the frog NMJ (Pinard *et al.*, 2003), may be responsible for the release of eCBs under physiological conditions. Under certain circumstances, glutamate may act either with or without ACh. Further experiments are needed to resolve these possibilities.

### Conclusion

Given the apparent high density of CB_1_ receptors on motor nerve terminals (e.g. Fig. 1A) and the relatively robust physiological effects of CB_1_ agonists and antagonists (e.g. Figs 2, 3 and 5), we find it surprising that it has taken so long for a role to be discovered for eCBs at the vertebrate NMJ. This is even more surprising given the fact that it has been known for many years that exogenous cannabinoids reduce motor function in rats (Compton *et al.*, 1996; Romero *et al.*, 1996) and humans (Ashton, 1999). We hope that the results presented in this work will stimulate further investigation into the role of eCBs at the NMJ. In addition to providing a greater appreciation of the diversity of roles for eCBs, the results reported here indicate that the vertebrate NMJ, which is an excellent model synapse for investigating the cellular and molecular details of synaptic transmission, may be employed to great advantage in studying details of eCB physiology.

## Abbreviations

ACh: acetylcholine
ACPA: arachidonylcyclopropylamide
2-AG: 2-arachidonoylglycerol
AM 281: *N*-(piperidin-1-yl)-5-(4-iodophenyl)-1-(2,4-dichlorophenyl)-4-methyl-1H-pyrazole-3-carboxamide
carboxy-PTIO: 2-(4-carboxyphenyl)-4,4,5,5-tetramethylimidazoline-1-oxyl-3-oxide potassium salt
DGL: diacylglycerol lipase
eCB: endocannabinoid
EPP: end-plate potential
L-NAME: N_ω_-nitro-l-arginine methyl ester
mAChR: muscarinic acetylcholine receptor
MEPP: miniature end-plate potential
NMJ: neuromuscular junction
NO: nitric oxide
ODQ: 1H-[1,2,4]oxadiazolo[4,3-a]quinoxalin-1-one
PLC: phospholipase C
PSC: perisynaptic Schwann cell
RHC-80267: 1,6-bis-(cyclohexyloximinocarbonylamino)-hexane
U-73122: 1-[6-[[(17b)-3-methoxyestra-1,3,4(10)-trien-17-yl]amino]hexyl]-1H-pyrrole-2,5-dione
VDM 11: (5Z,8Z,11Z,14Z)-*N*-(4-hydroxy-2-methylphenyl)-5,8,11,14-eicosatetraenamide
